# Tilting light’s polarization plane to spatially separate the ultrafast nonlinear response of chiral molecules

**DOI:** 10.1515/nanoph-2022-0802

**Published:** 2023-04-11

**Authors:** Laura Rego, Olga Smirnova, David Ayuso

**Affiliations:** Department of Physics, Imperial College London, SW7 2AZ London, UK; Universidad de Salamanca, 37008 Salamanca, Spain; Max-Born-Institute, Max-Born-Str. 2A, 12489 Berlin, Germany; Technische Universität Berlin, 10623 Berlin, Germany

**Keywords:** attosecond physics, high harmonic generation, ultrafast chiral spectroscopy

## Abstract

Distinguishing between the left- and right-handed versions of a chiral molecule (enantiomers) is vital, but also inherently difficult. Traditional optical methods using elliptically/circularly polarized light rely on linear effects which arise beyond the electric-dipole approximation, posing major limitations for ultrafast spectroscopy. Here we show how to turn an ultrashort elliptical pulse into an efficient chiro-optical tool: by tilting its polarization plane towards its propagation direction. This forward tilt can be achieved by focusing the beam tightly, creating structured light which exhibits a nontrivial polarization pattern in space. Using state-of-the-art computational modelling, we show that our structured field realizes a near-field interferometer for efficient chiral recognition that separates the nonlinear optical response of left- and right-handed molecules in space. Our work provides a simple, yet highly efficient, way of spatially structuring the polarization of light to image molecular chirality, with extreme enantio-efficiency and on ultrafast time scales.

## 1 Introduction

Chirality plays key roles in nature, from particle physics to biology. Something is chiral if it is different from its mirror image, with our hands being the typical example. The left- and right-handed versions of a chiral molecule are called enantiomers. Their handedness is essential in molecular recognition, and thus enantio-discrimination is vital. But it is also hard, as opposite enantiomers behave identically unless they interact with another chiral ‘object’, e.g. another chiral molecule or chiral light.

Modern laser technology creates exciting opportunities for imaging chirality, providing access to the natural temporal and spatial scales of molecules, with sub-femtosecond and sub-Angstrom resolution [1]. Yet, imaging the 3D chiral currents governing enantio-sensitive photo-chemistry is still very challenging, as natural chiral light is ill-suited for this purpose. Indeed, circularly polarized light is chiral only beyond the electric-dipole approximation, and thus it produces weakly enantio-sensitive signals, usually below 0.1% [2].

This limitation can be overcome by creating locally chiral fields [3–9], where the electric-field vector draws a chiral Lissajous figure in time, or by analysing enantio-sensitive vectorial observables [10–25] which do not rely on light’s chirality [26, 27]. This includes the photoelectron current orthogonal to the polarization plane of circularly [16–24] or elliptically [25] polarized light, which gives rise to the forward/backward asymmetry in photoelectron circular/elliptical dichroism. This current has opposite direction in opposite enantiomers and it is driven by electric-dipole interactions. However, if one seeks to record an equivalent asymmetry via nonlinear excitation with an elliptical field in an all-optical setup, measuring the radiation emitted by the induced polarization, they will encounter a fundamental limitation: this asymmetry is not in the right direction.

Let us consider a laser field **E** = *E*_0_(**u**_*x*_ + *iɛ* **u**_*y*_)e^−i*ωt*+**k**⋅**z**^ with ellipticity |*ɛ|* < 1. The nonlinear polarization induced in isotropic chiral media [14] is $P=P_{x}u_{x}+P_{\varepsilon }u_{y}+P_{c}^{L/R}u_{z}$ , where **u**_*x*_, **u**_*y*_ and **u**_*z*_ are the laboratory frame unitary vectors. The in-plane components *P*_*x*_ and *P*_*ɛ*_ are *achiral*: within the electric-dipole approximation, they are identical in left- and right-handed molecules. The out-of-plane component $P_{c}^{L/R}$ is *chiral*: it is exclusive of chiral media and has equal intensity but opposite phase in opposite enantiomers, $P_{c}^{L}=-P_{c}^{R}$ . However, $P_{c}^{L/R}$ is completely invisible in the typical macroscopic harmonic signal, as it is parallel to the laser propagation direction **u**_*z*_. For this reason, the recent chiral high harmonic generation (HHG) works with elliptical [28] and two-colour [29–31] fields relied on magnetic interactions.

Here we show how, by tilting the plane of polarization of the laser field towards its propagation direction, we can rotate the chiral component of the induced polarization, making it visible in the macroscopic far-field signal. Our proposal exploits the potential of structuring the polarization [32–36] of ultrashort pulses to realize an enantio-sensitive interferometer that separates the nonlinear response of opposite molecular enantiomers in space.

## 2 Proposed optical setup

**Figure 1:**
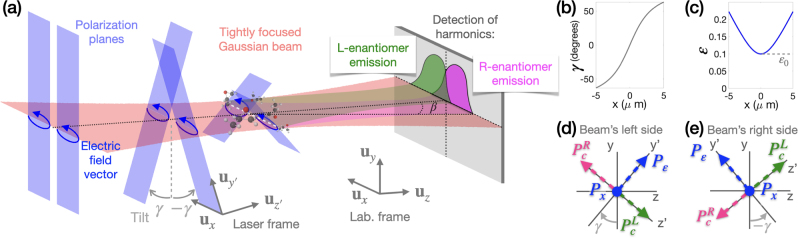
Tilting the plane of light’s polarization. (a) A laser field with elliptical polarization (blue arrows) and a Gaussian profile (light pink) acquires a forward polarization tilt upon tight focusing. The polarization of the laser at each spatial point is contained in a 2D plane (blue rectangles) and its tilt angle *γ* varies spatially, being maximum at the focus and having opposite signs at opposite sides of the beam’s axis. This tilt angle dictates the rotation of the laser frame with respect to the laboratory frame. The nonlinear optical emissions from the L enantiomer (green) and the R enantiomer (dark pink) are spatially separated in the detector, where they are recorded as a function of the emission angle (divergence) *β*. (b, c) Tilt angle *γ* (b) and total ellipticity *ɛ* (c) as functions of the transverse coordinate *x* for a beam’s waist of *W* = 2.5 μm. (d, e) Schematic representation of the polarization induced in randomly oriented chiral molecules at each side of the beam’s axis, see Eq. (2). Note that the chiral component of the induced polarization has opposite orientation in opposite molecular enantiomers.

We consider an ultrashort and tightly focused Gaussian beam with elliptical polarization, where the pulse duration is only a few cycles, and the beam waist is only a few times the wavelength. Such tight focusing creates a strong longitudinal electric-field component [37], along the propagation direction, which has opposite sign at opposite sides of the beam’s axis, see Figure 1(a) and Supplementary 1. The consequences of this new component are twofold. First, the plane of polarization rotates around the *x* axis, in opposite directions at opposite sides of the beam axis, and thus it stops being orthogonal to the propagation direction *z*. Second, the ellipticity increases. The field in one point in space can be written as

(1) $$E\left(t\right)=E_{0}\;a\left(t\right)\;\left[\cos\left(\omega t+\phi_{\text{CEP}}\right)u_{x}+\varepsilon sin\left(\omega t+\phi_{\text{CEP}}\right)u_{y^{\prime }}\right],$$

where *a*(*t*) is an envelope function, *ϕ*_CEP_ is the carrier-envelope phase (CEP) and **u**_*y*′_ = sin(*γ*)**u**_*z*_ + cos(*γ*)**u**_*y*_, with *γ* being the tilt (rotation) angle. Note that, thanks to the longitudinal component arising upon tight focusing, **u**_*y*′_ ≠ **u**_*y*_. That is, the minor component of the polarization ellipse of the laser is no longer orthogonal to the propagation direction *z*, see Figure 1(a). Importantly, both the tilt angle *γ* and the ellipticity *ɛ* are spatially structured, with *γ*(−*x*) = −*γ*(*x*) and *ɛ*(−*x*) = *ɛ*(*x*), see Figure 1(b, c). The tilt direction (sign of *γ*) is a protected quantity, robust with respect to experimental fluctuations, because it is connected to the spin-momentum locking [38], which relates the transverse photon spin to the propagation direction, see Supplementary 1.

The structured forward tilt of the laser polarization plane is a key aspect of our proposal. It leads to rotation of the nonlinear polarization induced in randomly oriented chiral media

(2) $$P\left(t\right)=P_{x}\left(t\right)u_{x}+P_{\varepsilon }\left(t\right)u_{y^{\prime }}+P_{c}^{L/R}\left(t\right)u_{z^{\prime }}.$$

That is, the chiral component of the induced polarization $P_{c}^{L/R}$ is tilted, no longer orthogonal to the propagation direction *z*, see Figure 1(d, e). In the laser reference frame, defined by the two laser polarization vectors, **u**_*x*_ and **u**_*y*′_, and **u**_*z*′_ = **u**_*x*_ × **u**_*y*′_, the three components of the induced polarization have exactly the same intensity in opposite enantiomers, and thus |**P**|_*L*_ = |**P**|_*R*_. The only enantio-sensitive quantity is the direction of the chiral component, $P_{c}^{L}=-P_{c}^{R}$ . To create an enantio-sensitive intensity, the chiral component needs to interfere with a reference signal. We can achieve this by projecting $P_{c}^{L/R}$ and one of the two achiral components (*P*_*x*_ and *P*_*ɛ*_) over a common axis that is in the right direction to produce a phase-matched macroscopic signal (orthogonal to *z*). This is exactly what the proposed optical setup does: by tilting the plane of polarization of light, we project *P*_*c*_ and *P*_*ɛ*_ over the common *y* axis, where they interfere:

(3) $$P_{y}\left(t\right)=P\left(t\right)\cdot u_{y}=P_{\varepsilon }\left(t\right)cos\left(\gamma \right)+P_{c}^{L/R}\left(t\right)\sin\left(\gamma \right).$$

Equation (3) shows that the tilt angle *γ* and the molecular handedness $\left(P_{c}^{L}=-P_{c}^{R}\right)$ control the relative sign between the achiral and chiral components of the induced polarization, creating an enantio-sensitive interferometer. Since *γ*(*x*) = −*γ*(−*x*), the chiral “arm” of our interferometer, $P_{c}^{L/R}\sin\left(\gamma \right)$ , has opposite phase at opposite sides of the beam axis. Thus, *P*_*y*_(*t*) is not only enantio-sensitive but also spatially structured.

We note that, in a long laser pulse, the chiral polarization component $P_{c}^{L/R}$ contains even harmonic orders, whereas the achiral components *P*_*x*_ and *P*_*ɛ*_ carry odd harmonic orders [14]. However, these selection rules relax as we reduce the pulse duration, broadening the spectral bandwidth, so the chiral and achiral components can efficiently overlap in the frequency domain. A recent proposal for chiral spectroscopy [39] showed that an ultrashort, linearly polarized, tightly focused, laser beam can produce harmonic light with enantio-sensitive polarization. Here, we realize a near-field interferometer that separates the nonlinear response of opposite molecular enantiomers in space, using an ultrashort laser field with a spatially structured polarization plane (Figure 1).

We have modelled the macroscopic electronic response of randomly oriented propylene oxide, fully accounting for the spatial structure of our field, using time-dependent density functional theory, see Supplement 1. We have considered these laser parameters: peak intensity *I* = 6 ⋅ 10^13^ W cm^−^^2^, incoming ellipticity *ɛ*_0_ = 0.1, focal diameter 5 μm, central wavelength *λ* = 780 nm and 7 fs full-width half-maximum (FWHM) of pulse duration. Such tight focusing and short duration conditions can be realized with current optical instrumentation [40]. Experiments would also benefit from using thin liquid microjets [41], where Gouy phase effects are negligible.

## 3 Numerical results

**Figure 2:**
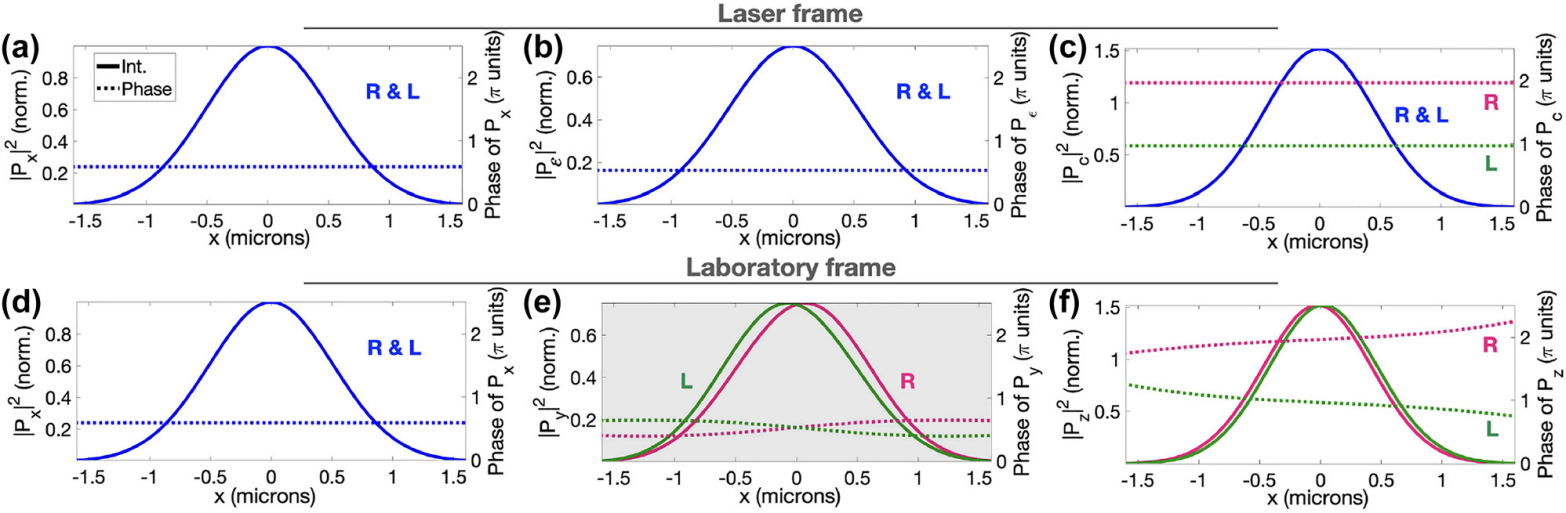
Near-field response of randomly oriented propylene oxide. Intensity (solid lines) and phase (dotted) at the 6th harmonic order in laser (a–c) and laboratory (d–f) reference frames for the R (pink) and L (green) enantiomers (non-enantio-sensitive curves are blue). The enantio-sensitive component that can be detected in the macroscopic far-field signal *P*_*y*_ is highlighted in grey shading (e). Laser parameters: *I* = 6 ⋅ 10^13^ W cm^−2^, *λ* = 780 nm, focal diameter 5 μm, *ɛ*_0_ = 0.1, pulse duration 7 fs (FWHM) and *ϕ*_*CEP*_ = 0.25*π*.

Figure 2 shows the amplitude and phase profiles of the induced polarization at frequency 6*ω* for *ϕ*_*CEP*_ = 0.25*π*, in the laser (Figure 2(a–c)) and laboratory (Figure 2(d–e)) reference frames. In the laser reference frame (Figure 2(a–c)), the three components of the induced polarization have the same intensity in opposite enantiomers. The molecular handedness is encoded in the phase of $P_{c}^{L/R}$ (Figure 2(c)). Thanks to our structured tilt, when projecting $P_{c}^{L/R}$ and *P*_*ɛ*_ on the laboratory-frame vectors, |*P*_*y*_|^2^ and |*P*_*z*_|^2^ become asymmetric with respect to the propagation axis and enantio-sensitive, see Figure 2(d–f). Note that the two reference frames are rotated around the *x* axis, and thus *P*_*x*_ is identical in both frames. Note also that, making *P*_*y*_ enantio-sensitive means that the intensity of phase-matched harmonic emission, proportional to |*P*_*x*_|^2^ + |*P*_*y*_|^2^, can become enantio-sensitive.

**Figure 3:**
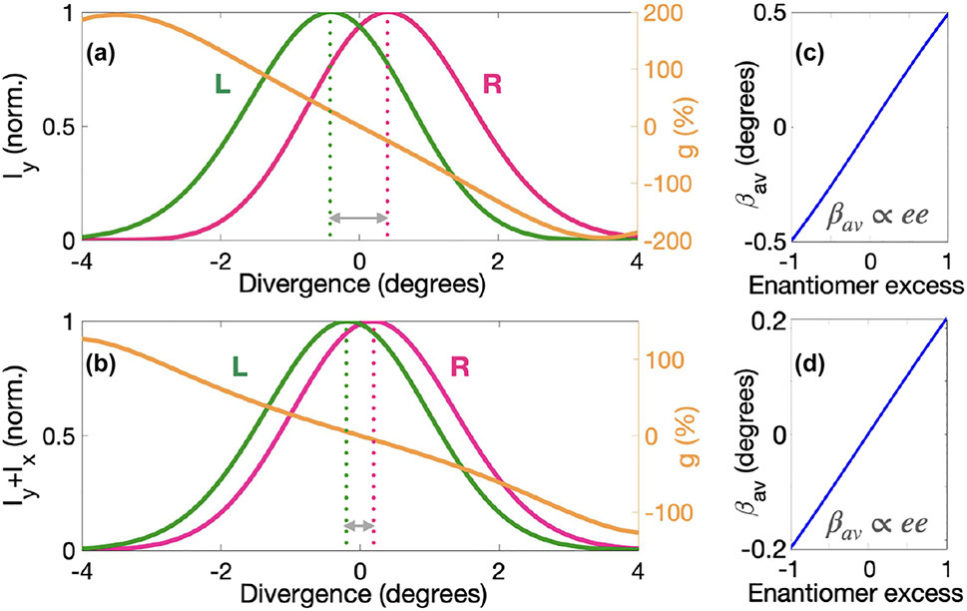
Enantio-sensitive harmonic detection. (a, b) Intensity of the *y*-polarized component *I*_*y*_ (a) of the radiation emitted from R (pink) and L (green) propylene oxide and total intensity *I* = *I*_*x*_ + *I*_*y*_ (b) at the 6th harmonic order, and dissymmetry factor (orange), as a function of the divergence angle, *β* = arctan (*x*/*z*), at the detector (see Figure 1). (c, d) Average divergence angle, *β*_*av*_, in *I*_*y*_ (c) and in *I* (d) as a function of the enantiomeric excess.

Figure 2(e) shows that the intensity profile of the *y*-polarized component of the induced polarization is asymmetric and different for left- and right-handed molecules. However, the most relevant asymmetry is in the phase of this quantity, which is also enantio-sensitive. As shown in Figure 2(e), the phase of the nonlinear response at frequency 6*ω* increases with *x* in the right-handed molecules, and it decreases in the left-handed molecules. This behaviour is similar for other harmonic frequencies (not shown). The phase profile shown in Figure 2(e) determines the propagation direction of the emitted harmonic light. For this choice of parameters, the left-handed molecules emit harmonic light preferentially to the left, whereas the right-handed molecules radiate preferentially to the right, see Figure 3.

The direction of harmonic emission is strongly enantio-sensitive when considering the intensity of the *y*-polarized component of the emitted harmonic light *I*_*y*_, generated by *P*_*y*_, see Figure 3(a). This component could be separated from the non-enantio-sensitive component *I*_*x*_ using a polarizer. However, our enantio-sensitive observable remains strong when considering the total intensity of the harmonic emission, *I*_*x*_ + *I*_*y*_, see Figure 3(b).

To quantify the degree of enantio-sensitivity in the macroscopic far-field signal, we use an angularly resolved definition of the dissymmetry factor *g* = 2(*I*_*L*_ − *I*_*R*_)/(*I*_*L*_ + *I*_*R*_), where *I*_*L*/*R*_ is the intensity of harmonic light emitted from the left-/right-handed enantiomer. The enantio-sensitivity approaches the ultimate efficiency limit (*g* = ±200%) in the *y*-polarized component of the emitted harmonic light (Figure 3(a)) but is also very strong when we consider the total macroscopic intensity (Figure 3(b)).

Note that, in our setup, changing the sign of the incoming ellipticity *ɛ*_0_ is equivalent to exchanging the molecular enantiomer. As a result, the dissymmetry factor is equivalent to the elliptical dichroism *g*_±_ = 2(*I*_+_ − *I*_−_)/(*I*_+_ + *I*_−_), where *I*_+/−_ is the intensity in the far field emitted when using a driving field with right/left elliptical polarization. Indeed, changing the sign of *ɛ*_0_ changes the sign of the achiral component of the induced polarization *P*_*ɛ*_cos(*γ*) (the achiral ‘arm’ of our near-field interferometer, see Eq. (3)) without modifying the chiral component *P*_*c*_sin(*γ*), whereas exchanging the molecular enantiomer changes the sign of *P*_*c*_ without affecting *P*_*ɛ*_. In both cases we are changing the sign of one of the two components without modifying the other, which leads to the same intensity profile in the far field—the only difference is in the phase of the emitted light, but this quantity is usually not measured experimentally and it does not enter the expressions of either the dissymmetry factor or the elliptical dichroism.

The proposed optical setup allows us to unequivocally determine the relative concentration of opposite enantiomers in mixtures, which is usually quantified via the enantiomeric excess, *ee* = (*C*_*R*_ − *C*_*L*_)/(*C*_*R*_ + *C*_*L*_), where *C*_*L*/*R*_ is the concentration of left-/right-handed molecules. The average divergence angle in the far-field harmonic intensity is approximately proportional to the enantiomeric excess, see Figure 3(c, d).

**Figure 4:**
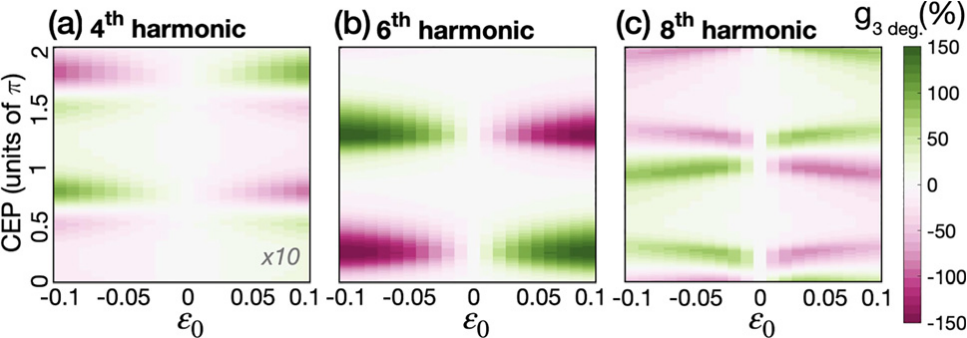
Maximizing the enantio-sensitive response of propylene oxide. Dissymmetry factor at a divergence angle of 3° as a function of the CEP and *ɛ*_0_ at the 4th (a), 6th (b) and 8th (c) harmonic orders. Reversing the sign of *ɛ*_0_ results in a change of sign in *g* and is equivalent to changing the CEP by *π*.

We can control the enantio-sensitive direction of harmonic emission by adjusting the laser parameters. In particular, by adjusting the CEP and the incoming ellipticity, we can adjust both the amplitude and phase of the achiral component of the interferometer *P*_*ɛ*_cos(*γ*), achieving full control over the enantio-sensitive interference. Figure 4 shows the dissymmetry factor in the *y*-polarized component of the emitted light for harmonic orders 4th, 6th and 8th, at 3 degrees of divergence. As expected, the values of the CEP and *ɛ*_0_ that maximize the enantio-sensitive response are different for different harmonic numbers, reflecting the fact that the relative amplitude and phase between the achiral (*P*_*ɛ*_) and chiral $\left(P_{c}^{L/R}\right)$ components of the induced polarization are frequency dependent.

Note that the proposed field is not chiral locally: at each point in space, the polarization of the electric field is confined to a 2D plane. The field becomes chiral only when we include its propagation direction. One could think that, in this scenario, the enantio-sensitive response of the medium must rely on weak magnetic or quadrupole interactions, as in traditional methods relying on the chirality of elliptically/circularly polarized light, but we have shown that this is not necessarily the case. Here, the propagation vector plays the role of a ‘chiral observer’ [27], dictating which components of the induced polarization can generate a phase-matched radiation that propagates to the detector and which cannot. It acts as a near-field polarizer, projecting two components of the induced polarization onto a common axis, where they efficiently interfere thanks the short pulse duration.

## 4 Conclusions

The proposed optical method realizes a near-field interferometer for efficient enantio-discrimination, which enables measuring the enantiomeric excess in mixtures. Our approach relies on a very special property of chiral molecules: their capability to turn in-plane rotation of the polarization of the electric-field vector of light into an out-of-plane molecular response. This property is at the core of efficient methods for chiral discrimination driven by purely electric-dipole interactions [26, 27]. While this molecular response remains hidden in the macroscopic optical signal when using conventional laser beams, here we have shown how, by tilting light’s polarization plane, we can convert it into a left/right asymmetry in the far-field signal. Thus, our work finds an interesting analogy with photo-electron circular dichroism [16–25], where a somewhat similar forward/backward asymmetry is recorded in the photo-electron angular distributions.

Because of the ultrafast nature of the nonlinear interactions responsible for the enantio-sensitive response, our method seems to be ideally suited for monitoring enantio-sensitive chemical reactions in real time, with sub-femtosecond temporal resolution. Furthermore, the enantio-sensitive direction of emission is a molecule-specific quantity, and thus our proposal creates new opportunities for developing molecular markers of enantio-sensitive chemical dynamics.

The strong longitudinal electric-field component that arises when we focus a laser beam tightly is a key ingredient of our proposal: it tilts the polarization plane of the elliptically polarized wave. Interestingly, such longitudinal fields also arise naturally in optical nanofibres and other nano-photonic structures [42], where light is confined in one or two spatial dimensions. Therefore, this work opens exciting opportunities for tilting light’s polarization using nano-photonic structures, and for developing nano-devices for highly sensitive chiral spectroscopy.

## Supplementary Material

Supplementary Material Details
