# Supplementary material for: Tilting light’s polarization plane to spatially separate the ultrafast nonlinear response of chiral molecules
Source: Nanophotonics. 2023 Apr 11;12(14):2873–9. doi: 10.1515/nanoph-2022-0802 (PMC11585975; doi:10.1515/nanoph-2022-0802)
Supplement: Supplementary file 1 — Supplementary Material Details [file j_nanoph-2022-0802_suppl_001.pdf]

Tilting light's polarization plane to spatially separate the ultrafast nonlinear response of chiral molecules SUPPLEMENTAL 1

LAURA REGO[1,2], OLGA SMIRNOVA[3,4], DAVID AYUSO[1,3]

[1] Department of Physics, Imperial College London, SW7 2AZ London, United Kingdom
[2] Universidad de Salamanca, 37008 Salamanca, Spain
[3] Max-Born-Institut, Max-Born-Str. 2A, 12489 Berlin, Germany
[4] Technische Universität Berlin, 10623 Berlin, Germany

1. ORIGIN OF THE ELECTRIC FIELD'S POLARIZATION TILT

Let us write the 2D polarization of the laser field considered in this work (Eq. 1 in the main text) in terms of the laboratory-frame vectors:

$$\mathbf{E}(t) = a(t)\ \Re\{(E_x\mathbf{u}_x + E_y\mathbf{u}_y + E_z\mathbf{u}_z)e^{-i\omega t - i\phi_{\text{CEP}}}\}, \tag{S1}$$

where $E_x = E_0$ is the strong-field component, $E_y = i\varepsilon_0 E_x$ is the incoming elliptical component, and E_z is the longitudinal component, parallel to the propagation direction z, which arises due to tight focusing [1, 2]. This component can be derived from Maxwell's equations, and it is given by:

$$E_z = \frac{i}{k}\left(\frac{\partial E_x}{\partial x} + \frac{\partial E_y}{\partial y}\right). \tag{S2}$$

In standard Gaussian beams, the weak spatial modulation of E_x and E_y leads to vanishing E_z. However, E_z becomes strong when light is confined in space, as it happens in a tightly focused beam. In a weakly elliptical field, with $E_x >> E_y$, the contribution from $\partial E_y/\partial y$ (second term in Eq. S2) is negligible, and we have

$$E_z = -i\frac{2x}{W^2 k}E_x. \tag{S3}$$

Note that E_z has opposite phase at opposite sides of the Gaussian beam axis due to the change of sign of $\partial E_x/\partial x$ at $x = 0$, see Eq. S2, whereas the phase of E_x and E_y does not change. As a result, the field acquires a non-trivial polarization pattern in space. Indeed, E_z and E_y are in phase at the left side of the beam ($x < 0$) and out of phase at the right side ($x > 0$), whereas E_z and E_x have a phase delay of $\pm\pi/2$. As a result, the plane of polarization of the driving field rotates around the x axis, i.e. it tilts towards the propagation direction, in opposite directions at opposite sides of the propagation axis.

Note that the tilt direction (the sign of γ) is connected to the so-called spin-momentum locking [3], which means that the direction of the transverse photon spin arising upon tight focusing is univocally determined by the direction in which the wave propagates. Therefore, the tilt direction is also a protected quantity, locked to the propagation direction of the wave and to the sign of the incoming ellipticity, which makes our approach robust with respect to possible experimental fluctuations.

2. CONTROL OVER THE LASER POLARIZATION

Here we describe how we can control the total ellipticity ε and tilt angle γ of the driving field by adjusting the beam waist W and incoming ellipticity ε_0. By comparing Eq. 1 in the main text with Eqs. S1 and S3, it is straightforward to see that the total ellipticity upon tight focusing in Eq. 1 is given by

$$\varepsilon = \frac{\sqrt{E_y^2 + E_z^2}}{iE_x} = \sqrt{\varepsilon_0^2 + [2x/(W^2k)]^2}, \tag{S4}$$

and the forward tilt angle is

$$\gamma = \arctan\left(\frac{E_z}{E_y}\right) = \arctan\left(\frac{-2x}{\varepsilon_0 W^2 k}\right). \tag{S5}$$

Eqs. S4 and S5 show how, by controlling W and ε_0, we can efficiently tailor the polarization tilt of the driving field. Fig. S1 in section 6 shows the values of ε and γ obtained for different combinations of W and ε_0.

3. ORIENTATIONAL AVERAGING OF THE LASER-INDUCED POLARIZATION

The induced polarization in the medium of randomly oriented propylene oxide molecules was calculated by averaging over the contribution from different molecular orientations:

$$\mathbf{P}(t,x) = \frac{1}{8\pi^2}\int_0^{2\pi}\int_0^{\pi}\int_0^{2\pi} \mathbf{P}_{\phi\theta\chi}(t,x)\ \sin(\theta)\ d\phi\ d\theta\ d\chi, \tag{S6}$$

where ϕ, θ and χ are the three Euler angles and $\mathbf{P}_{\phi\theta\chi}$ is the polarization response of a particular molecular orientation in the laboratory frame. We used the Lebedev quadrature [4] of order 11 (50 points) to integrate over ϕ and θ. For each Lebedev point, the polarization of the electric-field vector of the laser field was defined in a way that its strong-field component pointed in the same direction, which allowed us to reach convergence in χ using 4 points via trapezoidal numerical integration.

4. SINGLE-MOLECULE RESPONSE

The light-induced polarization was evaluated using real-time time-dependent density functional theory Octopus [5–8]. We used the local-density approximation [9–11] to account for electronic exchange and correlation effects, and the averaged-density self-interaction correction [12] to account describe the long-range behaviour of the electron density. The 1s orbitals of the heavier atoms (carbon and oxygen) are barely affected by the laser field, and they were described using pseudo-potentials. The Kohn-Sham orbitals and the electron density were expanded into a uniform real-space grid of points separated by 0.4 a.u. enclosed in a sphere of radius $R = 42$ a.u., and we used a complex absorbing potential with width 20 a.u. and height -0.2 a.u. to avoid unphysical reflexions of the electron density.

5. MACROSCOPIC CALCULATION: FAR-FIELD IMAGE

The intensity of HHG in the far field was calculated using the Fraunhofer diffraction equation:

$$F_\xi(\beta, N) \propto \int_{-\infty}^{\infty} \frac{d^2}{dt^2} P_\xi(x,N) e^{-i\frac{N\omega}{c}x\sin\beta} dx, \tag{S7}$$

where $\xi = x, y$, P_x and P_y are the non-enantio-sensitive and enantio-sensitive components of the induced polarization as a function of the transverse coordinate x in the near field in the frequency domain, N is the harmonic number, β is the horizontal divergence angle, c is the speed of light in vacuum, and ω is the fundamental frequency.

Note that, since the main component of the laser field is polarized in the x direction, and thus the longitudinal component only varies with E_x, this is the relevant coordinate to perform the macroscopic calculation. Due to the Gaussian profile of the driving field, the structure of the laser's polarization is the same for every value of y, and only its amplitude changes (following a Gaussian decay). For the harmonics below the ionization potential (such as the ones considered here) we can consider that the phase of the induced polarization does not depend on the laser's local amplitude. Thus $P_\xi(x,y,N) \propto P_\xi(x, y=0, N)$, which results in $F_\xi(\beta,\theta,N) \propto F_\xi(\beta, \theta = 0, N)$, being θ the vertical divergence angle, which means that the far-field harmonics conserve the same structure along θ. On the other hand, we consider that the target is very thin, to avoid fast phase variations of the driving field along z due to the Gouy phase, so propagation effects in the z direction are negligible.

6. CONTROL OVER THE LASER POLARIZATION

The total ellipticity ε and tilt angle γ of the driving laser field considered in the main text can be controlled by adjusting the beam waist W and the incoming ellipticity ε_0. Fig. S1a shows the spatial profile of ε along the transverse coordinate x, for different values of W and ε_0. Reducing W, i.e. making the beam more tightly focused, leads to a stronger longitudinal component, and thus to larger values of both ε and γ. The modulation of ε with ε_0 is rather trivial: increasing the incoming ellipticity leads to stronger total ellipticity. However, because the strength of the longitudinal component does not depend on ε_0 (for small ε_0), the tilt angle γ decreases when increasing ε_0. This is shown in Fig. S1b, which presents the values of γ as a function of ε for different values of ε_0.

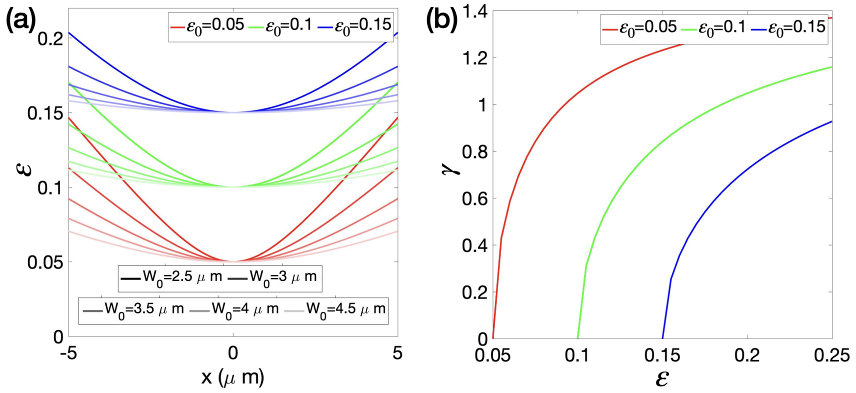

Fig. S1. **a,** Total ellipticity ε as a function of the transverse coordinate x for different values of the incoming ellipticity ε_0 and beam waist W. **b,** Forward tilting angle γ as a function of the total ellipticity ε for different values of ε_0.

7. ANGLE-INTEGRATED ENANTIO-SENSITIVE RESPONSE

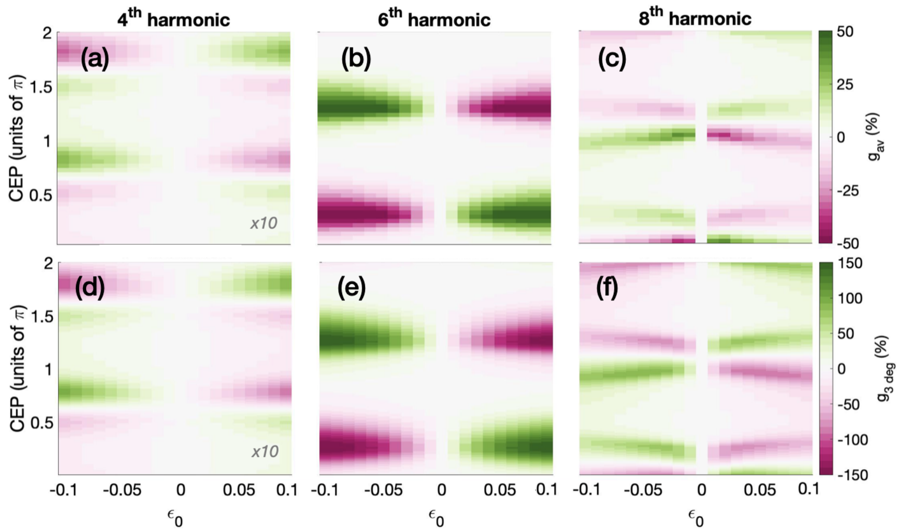

Fig. S2. Spatially averaged dissymmetry factor g_{av} (Eq. S8) as a function of the CEP and ε_0 in the light emitted at the 4th (**a**), 6th (**b**), and 8th (**c**) harmonic frequencies, see Fig. 2 of the main text for laser parameters. For comparison with the spatially resolved dissymmetry factor, see Figs. 3 and 4 of the main text.

Here we show how the enantio-sensitive response of the chiral molecules remains strong upon spatial integration. The dissymmetry factor $g(\beta)$ defined in the main text is an angularly resolved

quantity which depends on the divergence angle β, see Figs. 3a-b. We can define a spatially integrated quantity simply by weighting $g(\beta)$ by the intensity $I(\beta)$ and integrating over β. Since g has opposite signs at each side of the beam's axis, i.e. $g(\beta) = -g(-\beta)$, we restrict the integration to positive values of β, so the spatially averaged dissymmetry factor is calculated as:

$$g_{av} \quad = \quad \frac{\int_0^\infty g(\beta)\, I(\beta) d\beta}{\int_0^\infty I(\beta) d\beta}. \tag{S8}$$

Fig. S2 shows the average dissymmetry factor for the 4th, 6th, and 8th harmonic orders. Note that g_{av} keeps the sign of g for $\beta > 0$, see Fig. 4 of the main text. Indeed, the modulation of g_{av} with the CEP and ϵ_0 is qualitatively identical to that of g. Importantly, the degree of enantiosensitivity remains strong upon spatial integration, with the values of g_{av} being approximately three times smaller than the values of g at a divergence angle of 3 degrees (Fig. 4 of the main text).

REFERENCES

1. M. Lax, W. H. Louisell, and W. B. McKnight, "From maxwell to paraxial wave optics," Phys. Rev. A **11**, 1365–1370 (1975).
2. K. Y. Bliokh and F. Nori, "Transverse and longitudinal angular momenta of light," Phys. Reports **592**, 1 – 38 (2015). Transverse and longitudinal angular momenta of light.
3. P. Lodahl, S. Mahmoodian, S. Stobbe, A. Rauschenbeutel, P. Schneeweiss, J. Volz, H. Pichler, and P. Zoller, "Chiral quantum optics," Nature **541**, 473–480 (2017).
4. V. I. Lebedev and D. N. Laikov, "A quadrature formula for the sphere of the 131st algebraic order of accuracy," Doklady Math. **59**, 477–481 (1999).
5. N. Tancogne-Dejean, M. J. T. Oliveira, X. Andrade, H. Appel, C. H. Borca, G. Le Breton, F. Buchholz, A. Castro, S. Corni, A. A. Correa, U. De Giovannini, A. Delgado, F. G. Eich, J. Flick, G. Gil, A. Gomez, N. Helbig, H. Hübener, R. Jestädt, J. Jornet-Somoza, A. H. Larsen, I. V. Lebedeva, M. Lüders, M. A. L. Marques, S. T. Ohlmann, S. Pipolo, M. Rampp, C. A. Rozzi, D. A. Strubbe, S. A. Sato, C. Schäfer, I. Theophilou, A. Welden, and A. Rubio, "Octopus, a computational framework for exploring light-driven phenomena and quantum dynamics in extended and finite systems," The J. Chem. Phys. **152**, 124119 (2020).
6. X. Andrade, D. Strubbe, U. De Giovannini, A. H. Larsen, M. J. T. Oliveira, J. Alberdi-Rodriguez, A. Varas, I. Theophilou, N. Helbig, M. J. Verstraete, L. Stella, F. Nogueira, A. Aspuru-Guzik, A. Castro, M. A. L. Marques, and A. Rubio, "Real-space grids and the octopus code as tools for the development of new simulation approaches for electronic systems," Phys. Chem. Chem. Phys. **17**, 31371–31396 (2015).
7. A. Castro, H. Appel, M. Oliveira, C. A. Rozzi, X. Andrade, F. Lorenzen, M. A. L. Marques, E. K. U. Gross, and A. Rubio, "octopus: a tool for the application of time-dependent density functional theory," physica status solidi (b) **243**, 2465–2488 (2006).
8. M. A. Marques, A. Castro, G. F. Bertsch, and A. Rubio, "octopus: a first-principles tool for excited electron–ion dynamics," Comput. Phys. Commun. **151**, 60 – 78 (2003).
9. P. A. M. Dirac, "Note on exchange phenomena in the thomas atom," Math. Proc. Camb. Philos. Soc. **26**, 376–385 (1930).
10. F. Bloch, "Bemerkung zur elektronentheorie des ferromagnetismus und der elektrischen leitfähigkeit," Zeitschrift für Physik **57**, 545–555 (1929).
11. J. P. Perdew and A. Zunger, "Self-interaction correction to density-functional approximations for many-electron systems," Phys. Rev. B **23**, 5048–5079 (1981).
12. C. Legrand, E. Suraud, and P.-G. Reinhard, "Comparison of self-interaction-corrections for metal clusters," J. Phys. B: At. Mol. Opt. Phys. **35**, 1115–1128 (2002).
